# HOW CAN PATIENT PORTALS SUPPORT CAREGIVERS PROVIDING END-OF-LIFE CARE?: A MIXED-METHODS STUDY

**DOI:** 10.1093/geroni/igac059.842

**Published:** 2022-12-20

**Authors:** Jennifer Dickman Portz, John David Powers, Megan Baldwin, Kathy Gleason, Rebecca Boxer, Ted Palen, Elizabeth Bayliss

**Affiliations:** University of Colorado Anschutz, Aurora, Colorado, United States; Kaiser Permanente Colorado, Denver, Colorado, United States; Kaiser Permanente Colorado, Denver, Colorado, United States; Kaiser Permanente Colorado, Denver, Colorado, United States; Kaiser Permanente Colorado, Denver, Colorado, United States; Kaiser Permanente Colorado, Denver, Colorado, United States; Kaiser Permanente Colorado, Denver, Colorado, United States

## Abstract

Patient portal design is rarely centered around patient caregivers—family members, friends and other designated persons who may access these digital tools for the patient’s health needs. This study examines: (a) portal features used by caregivers during their loved-one’s end-of-life; and (b) caregiver perceptions regarding the use of patient portals for caregiving near the end-of-life.Using sequential mixed-methods, we conducted a retrospective cohort analysis of caregiver proxy (N=137) use from 2016-2019 during their loved-ones’ (N=5,284) last 12 months of life and interviews with (N=31) 16-former and 15-current caregiver proxies. Caregiver proxy portal use in this population was lower than expected; however, caregivers who used the portal as proxies found value in tools for caregiving near the end-of-life. To leverage the patient portal to better support caregivers, future strategies should target caregiver portal adoption, awareness of beneficial portal tools, and the addition of caregiver specific resources and education.

